# Challenges and Opportunities with Empowering Baby Boomers for Personal Health Information Management Using Consumer Health Information Technologies: an Ecological Perspective

**DOI:** 10.3934/publichealth.2014.3.160

**Published:** 2014-09-02

**Authors:** Cynthia M. LeRouge, Donghua Tao, Jennifer Ohs, Helen W. Lach, Keri Jupka, Ricardo Wray

**Affiliations:** 1 College for Public Health and Social Justice, Department of Health Management and Policy, Saint Louis University, Saint Louis, MO 74444, USA; 2 Medical Center Library, Saint Louis University, Saint Louis, MO 74444, USA; 3 College of Arts and Sciences, Department of Communication, Saint Louis University, Saint Louis, MO 74444, USA; 4 School of Nursing, Saint Louis University, Saint Louis, MO 74444, USA; 5 Department of Behavioral Science and Health Education, College for Public Health and Social Justice, Saint Louis University, Saint Louis, MO 74444, USA

**Keywords:** Baby Boomers, consumer health information technology, personal health information management, ecological model, conceptual model

## Abstract

“Baby Boomers” (adults born between the years of 1946 and 1964) make up the largest segment of the population in many countries, including the United States (about 78 million Americans) [1]. As Baby Boomers reach retirement age and beyond, many will have increasing medical needs and thus demand more health care resources that will challenge the healthcare system. Baby Boomers will likely accelerate the movement toward patient self-management and prevention efforts. Consumer Health Information Technologies (CHIT) hold promise for empowering health consumers to take an active role in health maintenance and disease management, and thus, have the potential to address Baby Boomers' health needs. Such innovations require changes in health care practice and processes that take into account Baby Boomers' personal health needs, preferences, health culture, and abilities to use these technologies. Without foundational knowledge of barriers and opportunities, Baby Boomers may not realize the potential of these innovations for improving self-management of health and health outcomes. However, research to date has not adequately explored the degree to which Baby Boomers are ready to embrace consumer health information technology and how their unique subcultures affect adoption and diffusion. This position paper describes an ecological conceptual framework for understanding and studying CHIT aimed at satisfying the personal health needs of Baby Boomers. We explore existing literature to provide a detailed depiction of our proposed conceptual framework, which focuses characteristics influencing Baby Boomers and their Personal Health Information Management (PHIM) and potential information problems. Using our ecological framework as a backdrop, we provide insight and implications for future research based on literature and underlying theories represented in our model.

## 1. Introduction

Baby Boomers have growing health demands that will challenge the US system. Adults born between the years of 1946 and 1964 (Baby Boomers) make up the largest segment of the population in many countries, including the United States (about 78 million Americans) [1]. In 2011, the first Baby Boomers reached age 65; beginning what is called the “gray tsunami.” Many of these individuals will have increased medical needs as they age, and thus demand more health care resources than other segments of the population. Due to their sheer numbers, experts anticipate the need for a larger workforce who can care for this aging population, as well as ways to make care more efficient [2]. Reducing health care expenses and improving quality, along with strategies to prevent acute illness and improve health management are important [3].

With respect to prevention and management, there is an ever-shifting burden of care from provider to patient, particularly given current health policy and increasing pressure for providers to contain cost [4]. Baby Boomers, who have higher levels of education and active consumerism than previous generations, are likely to accelerate the movement toward patient self-management of chronic disease [5]. To effectively take on this responsibility, Baby Boomers will need efficient means of Personal Health Information Management (PHIM), which includes managing personal health information, and finding and using consumer health information. As a result, the need for better tools to support patient self-management of health and information will increase substantially.

Bodenheimer et al. contend self-management requires that patients accept the responsibility of managing their own conditions and solving their own problems by integrating information from providers and other available sources [6]. However, there are considerable PHIM challenges to connecting Baby Boomers to credible, up-to-date, easy-to-understand information about diet, lifestyle changes, medications and specific conditions. Some may not have the technical skills required to self-manage certain conditions (e.g. blood glucose monitoring) and obtain all the information needed to make health-related decisions. For example, in a systematic review, Goldzweig et al. [7], found lower use of patient portals for health information among ethnic minorities and those with low literacy. Other PHIM issues stem from the quality of available information. Information gathered from Internet-based sources varies in accuracy and is sometimes contradictory. Additionally, new systems are not always user friendly or easily accessible for patients. Effective PHIM relies on both individual and system level factors, including public and professional communication skills, requirements of public and private health systems, and situational demands. CHIT hold promise for empowering health consumers to take an active role in health maintenance and disease management, and thus assist in addressing Baby Boomers use of PHIM. Indeed, we propose that the quality of healthcare for aging Baby Boomers hinges as much on the development, promotion, and delivery of personal health information as it does on enhancing health care services. Such promising innovations require changes in health care practice and processes that need to take into account Baby Boomers' personal health needs, preferences, health culture, access and abilities to use these technologies [8]. Without foundational knowledge of barriers and opportunities, providers may not be able to help Baby Boomers realize the potential of these innovations for improving self-management of health and health outcomes. Understanding how CHIT could support Baby Boomers' health, health care, and PHIM has significant practice and policy implications, yet research on this population is limited.

The overarching mission of this conceptual paper is to advance understanding of the Baby Boomer population to better inform the study, design, and use of CHIT for their PHIM. It is our hope that this paper sparks thought and serves as a call to action for development of CHIT aimed at supporting Baby Boomers and their health self-management. The inherent complexities of such an area of research require an interdisciplinary approach that addresses the multi-dimensional socio-cultural, health informatics, information management, and communication dimensions of CHIT for PHIM. This approach is recommended by the National Academy of Sciences [9] and Institute of Medicine [2] to promote innovation and a holistic approach to complex, emerging areas of study. As such, the authors took an integrative approach and developed an interdisciplinary collaborative to examine CHIT and PHIM of Baby Boomers.

This position paper first presents a brief overview to set the foundation for an interdisciplinary, ecological approach to studying Baby Boomers' use of CHIT for PHIM. Subsequently, we present a detailed depiction of our ecological conceptual framework that focuses on the characteristics of the Baby Boomer population, health care needs and PHIM behaviors, and the potential role of CHIT for their PHIM. We then recap literature on each of the key model constructs (individual characteristics, CHIT use, PHIM behaviors, decision making, health behaviors, health outcome, and external ecological influences) as they relate to CHIT and Baby Boomers. In highlighting scholarship, we demonstrate where there is a dearth of present knowledge related to the individual constructs as well as gaps in the literature connecting them. Finally, we lay out propositions and direction for future research.

## 2. Materials and Methods

The Baby Boomer population approaches retirement and old age over the next 30 years. Through their life course and experiences, Baby Boomers have changed every facet of society [10]. Several factors related to health care have been identified as important in determining Baby Boomers' future impact on health care, including chronic disease and disability, their role as active consumers, and technology [11]. Understanding the health-related technology access, needs, preferences and abilities of this population is crucial for developing tools to promote healthy, successful aging. The proposed ecological model serves as a framework to begin to address this issue.

Ecological theory, at a broad level, posits that health and behavior are influenced at multiple levels, including individual, interpersonal, sociocultural, organizational and environmental, and that these influences interact with one another [12]. Ecological model includes an emphasis on environmental characteristics (e.g., community and workplace design), access to elements important to behaviors (e.g., healthy or unhealthy food, opportunities for physical activity), and the impact of technologies (e.g., the web and other media). Further, an ecological perspective demonstrates the intertwined nature of such factors. Past research has demonstrated the value of using an ecological perspective, as health behavior is influenced by a variety of interconnected factors, such that interventions to influence health decisions and behaviors are more effective when they include applications at multiple levels. For example, an ecological model helps explain the multiple and interacting levels of intervention important in addressing tobacco prevention [13]. Other research has demonstrated the limitations of obesity interventions solely considering the intrapersonal, interpersonal, and cognitive levels [5]. Ecological perspectives are particularly valuable when considering complex health situations such as health information seeking and health-related decision making. This perspective has also helped explore the varied resources from the health care system and community that support self-care in diabetes [3]. Additionally, Patrick, Intille & Zabinski explored the necessity of utilizing an ecological approach in order to advance the potential uses of the Internet for cancer communication [14]. Similarly, the Chronic Care Model [15] posited by Wagner effectively integrates consumer, provider, social support and health system components (such as clinical information systems) to enhance self-management support [16].

**Figure 1.**
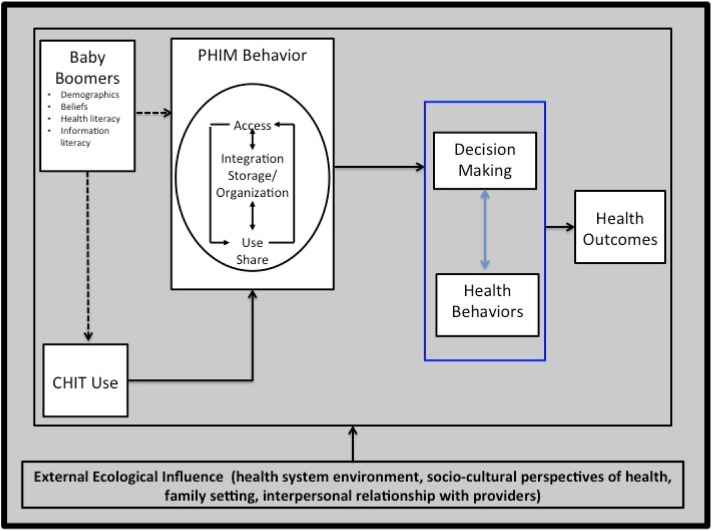
Ecological model of Baby Boomer personal health information management using consumer health information technologies (Ecological–Boomer PHIM using CHIT).

We frame our conceptual position as shown in Figure 1. We introduce this model to guide exploration of CHIT and PHIM among Baby Boomers that will inform future directions in research, practice and information technology design. The model posits that a combination of individual factors (demographics, beliefs, and health and information literacy) as well as CHIT use influence PHIM behaviors (access, integration, storage, organization, use and sharing) that in turn affect health decision making, health behaviors, and health outcomes (morbidity, mortality, quality of life and cost of health care). An ecological perspective places the above pathways of influence in a context of external or system factors, including interpersonal relationships, family systems, provider interactions, organizational contexts of health systems, socio-cultural factors, governmental policy, and technology.

We use this model as a conceptual framework to connect disparate pockets of knowledge in various disciplines to gain a better understanding of the use of CHIT by Baby Boomers for PHIM and set forth a research agenda.

## 3. Results-Conceptual model constructs

### 3.1. Baby Boomers

A variety of characteristics affect Baby Boomers' PHIM behaviors and CHIT use, and thus further impact their health care decisions and health behaviors. Baby Boomers include seventy-eight million people born following World War II. This group is now aging; the population over 65 is projected to double by 2030, reaching over 72 million [17]. Baby Boomers matured in a time of growth and change in the United States, and throughout their lives, the sheer size of this generation significantly impacted various facets of society [18] including health care.

A number of features of the Baby Boomer generation are important for understanding their PHIM and health behaviors. Baby Boomers are diverse in term of both race and ethnicity [19]. For example, Latinos make up 10% of this generation [20]. When it comes to personal well-being, Baby Boomers are conscious of youth, beauty and appearance, and seek to postpone old age [21],[22]. Regarding work life, compared to the generation immediately preceding them, currently older adults, Baby Boomers have more education and higher level jobs, and more women are working; they are the first to telecommute and are likely to work past the traditional retirement age of 65 [16]. Regarding home and family, Baby Boomers in the US are more geographically mobile than earlier generations and tend to live in the suburbs [19]. Baby Boomers have higher rates of separation and divorce, lower rates of marriage, and have fewer children, which will affect their finances and human resources in later life [19].

Compared to earlier generations, Baby Boomers appear to understand the need to be proactive in order to achieve long-term health and successful aging. Significant attention has been paid to prevention for this age group, which could have important implications for future health care costs. They are also more health conscious than their parents. However, while 90% of Baby Boomers report trying to exercise or eat healthier, many are not able to sustain these changes [23]. As active consumers, they may have higher expectations of the health care system in terms of being involved in their care and managing health problems [24]. In addition, it seems that many have adopted preferences for alternative medicine [25].

Existing literature provides few indications of how the life complexities and circumstances experienced by Baby Boomers interfere or aid with their use of technology for self-care, now or in the future. Various subgroups of the Baby Boomer population may have differing needs based on differing demographic characteristics such as age and gender. For example, there are greater health literacy problems among minority and immigrant groups [7], which influences health information consumption.

Baby Boomers have witnessed the explosive growth and convergence of communication and information technologies. This could provide opportunities for managing health. Research with a focused on further understanding the health culture(s) of this generation is urgently needed to help identify ways to make care more efficient, provide support for taking an active role in health, and assist with promoting health and managing chronic conditions through technology [26].

CHIT may include the Internet, social media (Web 2.0), mobile devices, and electronic personal health records, etc. Much of what is known about Baby Boomers' use of health technology is related to assisted living devices (e.g., personal emergency response systems, fall detection systems, switches, motion sensors, computer vision), rather than PHIM technologies [27],[28],[29]. Research shows that assistive living technologies may help keep many patients out of the hospitals and provide communication links with caregivers [30].

Studies of general information technology use indicate that most Baby Boomers are Internet, email, and social network users [31]. Use of such information and communication technologies may support consumers in managing their personal health information and making decisions regarding their healthcare. Indeed, CHIT is believed to have the potential to help older adults maintain quality of life and independence as they age [4]. Baby Boomers are predicted to be receptive to the use of technology to manage disease and disability [32]. However, research has not yet comprehensively addressed the type and nature of the PHIM technology to which Baby Boomers will respond best [32] and the impact of technology for PHIM.

Though a range of PHIM technologies exist, research tends to focus on Baby Boomers using the Internet and personal health records. As the Baby Boomers develop chronic diseases, the gap between their desire for information and physicians' ability to provide it is likely to increase. CHIT has the potential to narrow the gap. One study demonstrated that Baby Boomers using integrated personal health records shared with providers enhanced patients' ability to become active partners in their health care [33]. Technology that creates shared space for providers and Boomers to manage their health care has the potential to aid Baby Boomers' health management, but is relatively understudied.

Studies have shown that 60% of people using the Internet for health information seeking are over 40 years of age, of which Baby Boomers are part [34],[35]. A Pew research study found that ‘a majority of e-patients access user-generated health information with 41% having read a commentary/blog/newsgroup about a particular health or medical issue [35]. “Yet, the full potential of Internet for health promotion has not been met, especially for the Baby Boomer generation.” [36]. Though there is a dearth of evaluation and design studies of PHIM specified to Baby Boomers, studies do exist that provide insight regarding whether Baby Boomers seek specific health information on the Internet. For example, one study demonstrated that Baby Boomers aged 50–64 were just as likely as those 35–49 to look for cancer-related information on the Internet. It is also important to note that those searching for cancer information on the Internet felt that information was inadequate [37]. Results from a 1997 survey of rural patients indicated that only 1% of respondents used the Internet as their primary source to obtain drug information [38]. The explanation for these results is that direct access to sources of information about drugs (pharmacist, physician, drug packaging), is preferable, weakening the need to search for information on the Internet. These two studies suggest that inadequate information and information received elsewhere impact the use of health-related websites. Determining the specific types of health information Baby Boomers would like to see on the web and their assessment of health-related sites would be productive. In addition, exploring the need, design, and impact of Baby Boomers' use of web sites to find information about specific health issues, like cancer or diabetes, is warranted.

Smart phones also have the potential to connect Baby Boomers to Internet sites. Additionally, smart phone applications developed for PHIM are growing dramatically. In addition, the web 2.0 tools and social media sites, such as Facebook and YouTube, have become popular avenues to share health information with fellow health consumers and providers to seek online health support [45],[46]. And Reassen and colleagues found that 27% of respondents in their cross-country European study had participated in online forums or self-help groups [32]. Advantages of using these online tools include protecting users' identity, fast spread of information, and one-stop shopping for information on topics of interest, such as symptoms, side effects, specific treatment, healthcare provider recommendations, prognosis, and self-care after treatment [47]. The quality and usability of such applications for Baby Boomers' health needs has not received adequate research attention.

Baby Boomers may also find benefit in PHIM in their role as informal health care giver (supporting network) for older children in the home and/or aging parents. Koch noted that family members, relatives, and informal caregivers are important resources and targeted customers for technology-based products designed to aid older adults in managing their health [39].

PHIM technologies may be particularly useful for the self-motivated health consumer who is willing and able to interact with technologies. Tailored design may contribute to motivation and adoption of PHIM. Evaluation of design features needed to appropriately tailor sites to Baby Boomers has not received much attention; likewise, methods to achieving changes in health attitudes and beliefs through CHIT have also been under studied [36]. A noted exception is recent work that explores persuasive technologies, computing systems, devices, or applications designed to change a person's attitude or behavior, with the intention of improving health behaviors, reducing health care costs, and allowing aging adults to maintain independent living [40]. The persuasive design approach emphasizes understanding the user (i.e., Baby Boomers) for promoting attitude and behavior changes.

How Baby Boomers manage health information and garner support using CHIT is an evolving phenomenon. More information is needed to understand these complex interactions and design health information systems to meet their needs.

### 3.2. PHIM behavior

PHIM behavior is defined as “activities that support consumers' access, integration, organization, and use of their personal health information” [41]. Examples of such activities include creating personal health history, making lists to support health activities (e.g. medication, provider, and allergy lists) [41], aggregating health information from different sources, and setting health reminders. The following section provides a snapshot about Baby Boomers' health information seeking behaviors, health information organization behaviors, and health information use and sharing behaviors.

**Figure 2.**
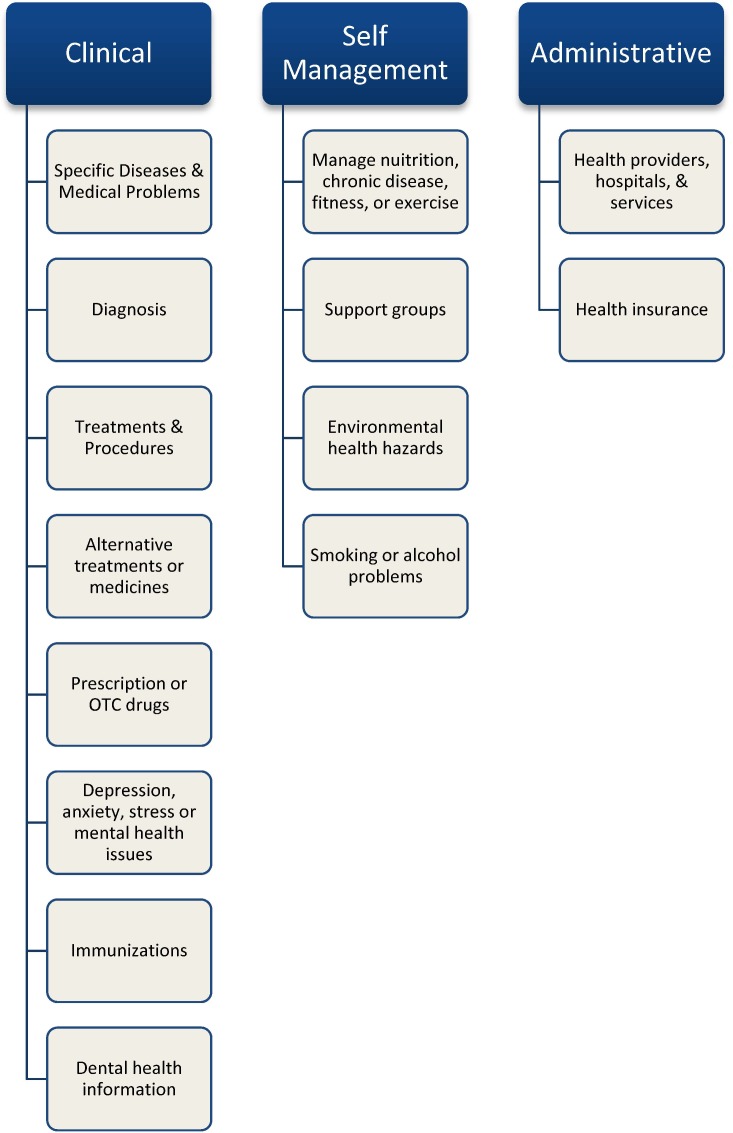
Frequently sought health information [48],[54],[55].

Baby Boomers seek different types of information based on their health status, stage of treatment and disease treatment phase. The health information most frequently sought by Baby Boomers is presented in Figure 2. Baby Boomers sought this information through a number of means. Some common sources include health care providers, health care insurers, individuals' social network members, and computer-based resources [42],[43].

At the stage of assessment and treatment, diagnosis and prognosis information is needed most; while patients are more interested in the self-care information after treatment [44]. According to Andreassen et al., “medical indicators of health, such as current diagnosis of long-term illness or disability and a high number of visits to the general practitioner, indicate a higher level of health-related use of the Internet” [32].

Baby Boomers search for health information to achieve a variety of goals: preparing for consultations with a health care provider, monitoring and assessing health, planning preventive or treatment actions, and making health-related decisions. These goals form the foundation for tasks, such as creating a health history, making lists of questions for a provider, integrating information, and establishing reminders [41],[45]. Furthermore, Baby Boomers with diseases or pursuing better health seek information to reduce health related anxiety and uncertainty, as well as to take control of their health to improve the quality of life [45],[46].

Baby Boomers' health information seeking behaviors (HISBs) are influenced by factors like age, gender, race/ethnicity, educational level, health status, medical history, family health history, financial and employment status, health beliefs, health literacy, and access to healthcare services, etc.[47],[48]. Generally speaking, female Baby Boomers, younger Baby Boomers, and Baby Boomers with higher education levels tend to actively seek health information [48]–[53]. In addition, Baby Boomers with family history and personal medical history prefer to go to the Internet as their first source of information over physicians [50].

The Pew Internet and American Life Project [54],[45] reported that information obtained online is more likely to affect treatment decisions, interactions with doctors, ability to cope with medical conditions, and diet and fitness regimens of individuals suffering from a disability or chronic condition compared to other users of online health information. Studies have also found that the level of involvement in health-related decisions is associated with health information seeking, education level, and patient-provider relationship. For example, active health information seekers have a higher level of involvement in decisions about health care and treatment [56]. Baby Boomers with higher education conceive their involvement as sharing the responsibility of decision making with the doctor [57]. Higher levels of education improve ability to verify the credibility of information and explore options beyond those presented in clinical consultations. In addition, patient-provider relationships and providers' interpersonal communication skills also influence Baby Boomers involvement in decision making [57]. Studies show that Baby Boomers who actively seek health information reported positive health assessments and the lowest occurrence of health risk factors [58]. Clearly, health information seeking behaviors and abilities are important for understanding Baby Boomer's health care decisions and health behaviors.

Information storage, organization, and integration are the activities and processes of filing, saving, labeling, classifying, and aggregating information either in paper or electronic format for the purpose of easy retrieval of stored information. Health information can be hard to store, organize, and integrate into one central resource due to being largely fragmented and scattered in multiple repositories and formats, and multiple types such as lab results, vital signs, provider information, and medication lists [41],[59]. Information about diagnosis and treatment is usually kept in the provider or hospital medical records. However, with more responsibility for self-management shifted to patients, Baby Boomers also need access to and organize their clinical health information, such as blood pressure or cholesterol levels. Additionally, Baby Boomers may want to monitor daily health behaviors, such as food consumption and exercise. How Baby Boomers store and organize their personal health information, especially associated with chronic disease management, has been rarely studied.

#### 3.2.1. Health information storage

Some studies show that Baby Boomers are willing to use computer-based programs to store personal health information [60],[61], such as PHRs, smart phones, and Personal Health Application (PHA). However, whether the health information stored in these information systems is organized in the way Baby Boomers prefer is unclear. Moen and Brennan [62] studied the storage strategies of Baby Boomers personal health information and labeled strategies with "just-in-time", "just-because", "just-in-case", and "just-at-hand” based on the location of the information and urgency in the need to retrieve it [62]. Understanding the specific practices, preferences, and strategies of Baby Boomers for storing and organizing their personal health information would provide information for designers and developers to create systems or tools that can better cater to Baby Boomers' needs [63] and would also suggest how CHIT can facilitate Baby Boomers' PHIM.

#### 3.2.2. Health information use and sharing

Baby Boomers live in an era wrought with information overload and conflict (e.g., whether to eat eggs or drink wine). Moreover, much of the health information available online suffers from inaccuracies or marketing bias (e.g., websites sponsored by pharmaceutical companies). Overwhelming and conflicting information from multiple resources cause consumers confusion about what recommendations to trust and follow, which can have negative implications. For example, Carpenter found that patients who encounter conflicting medication information are less adherent to their prescriptions [57], although the presence of a supportive physician may counteract this consequence [58]. Characteristics of Baby Boomer subgroups based on, for example, demographic information, education, socioeconomic status, health status, role as patients or caregiver, and beliefs about health and health services, and contexts within which they reside (e.g. living environment, social connections, technology environment, and availability of health services) affect consumers' preferences for using and trusting certain information sources [42]. Understanding such characteristics and contexts will aid in helping Baby Boomers overcome information challenges.

Baby Boomers' may share their health information with various audiences, including providers, researchers, members of their personal social network (e.g., children, siblings, friends, and other caregivers), insurance companies, health organizations and others. For example, some Baby Boomers may share health information with personal caregivers, such as adult children, to help them with healthcare decisions [75]. Concerns about privacy and confidentiality surface when Baby Boomers consider sharing their personal health information. Concerns might stem from trust with particular audiences or security protocols set in the systems [64]. Baby Boomers want to have options to control personal health information:

- What information they want to be shared
- With whom will they share
- When will sharing occur
- What is the purpose of sharing
- What are the best means for doing so? [65]

Studies found that Baby Boomers are more accepting of health information shared with others for research, providing care, or quality improvement than for health information shared for marketing purposes [66],[67]. Baby Boomers prefer to be asked permission before their health information is used for any purpose other than medical treatment; they would also like to know the organization and details of the research before sharing their health records [68]. De-identifying personal health information (e.g., remove name and address) reduces Baby Boomers' concerns of privacy and confidentiality [67],[68],[69]. Baby Boomers are less open to sharing sensitive information, such as sexually transmitted diseases, abortions, infertility, family medical history/genetic disorders, mental illness, drug/alcohol related incidents, lists of previous operations/procedures/dates and current medications [70]. Level of education, race and ethnicity, cultural background and employment status are strongly associated with these concerns. However, how Baby Boomers use and share their personal health information and how these use and share behaviors are affected are still under study. Understanding use and share behaviors will help better design CHIT tools and patient-provider communications, as well as overall health information management and exchange among different health systems and technology platforms.

### 3.3 Baby Boomers' decision making, health behaviors and outcomes

Decision making is a critical topic in health as it represents the intersection of the model precursors (individual characteristics, CHIT use and PHIM behaviors) with health behaviors and outcomes. Decision making is a reasoning process which can be rational or irrational, and can be based on explicit assumptions or tacit assumptions [71]. It is a cognitive process that leads to a choice among alternatives, even if the choice is not to act.

Health decision making has become an increasingly important area of research due to recent shifts toward patient-centered care. This shift in focus is reflected in an emphasis on informed or shared decision making. According to Street [72], “To more effectively participate in the decision-making process, patients should have some understanding of their health conditions, the risks/benefits associated with different treatment options, and an opportunity to integrate this information with their personal belief and value system”. As mentioned previously, existing literature provides few indications of how the life complexities and circumstances that Baby Boomers face interfere or aid their participation in health decision making and use of technology.

The health behaviors component consists of behavior patterns, actions and habits related to physical and mental health [73]. Behaviors within this definition can include medical service usage (e.g., physician visits, vaccination, screening), adherence with medical regimens (e.g., dietary, medication, management of chronic disease), and self-directed health behaviors (e.g., diet, exercise, meditation, smoking, alcohol consumption, management of chronic conditions) [74].

The final component of the model is to examine health outcomes, defined as the “end results of particular health care practices and interventions,” [75] and applies to individuals, groups or populations [76],[77]. Possible outcomes from the use of PHIM range from morbidity, mortality, and functional to health status, to quality of life and costs of care. Measures of these outcomes can be used to enhance health care decisions and improve quality of care [83]. Additionally, assessment of patient perceptions, such as satisfaction, patient understanding and ability to manage health issues are examined [78].

As we look at current Baby Boomers, questions emerge about their anticipated health needs and the impact of those needs on decision making, behaviors, and health outcomes. Baby Boomers have increasingly longer life expectancies and in some ways are healthier than the prior generation [79]. However, about 60 percent of Baby Boomers have already been diagnosed with at least one chronic medical condition, such as arthritis, diabetes, heart disease, obesity, osteoporosis, hypertension and depression.[6],[80]. These conditions will require regular medical checkups, prescription medications and/or dietary management, and have the potential to lead to disability. Researchers have noted that prior trends of declining disability among older adults are reversing, and Baby Boomers are not doing better than earlier cohorts [81], but have higher rates of chronic disease, disability and lower self-rated health than the previous generation [82].

Potential reasons for these health needs and issues are many. Competing demands of work and family may influence attention to health promoting behaviors. Currently, many Baby Boomers are caring for both children and aging parents, hence their label as the “Sandwich Generation” [83]. Baby Boomers incur the financial and emotional burden of caring for multiple parents and children, which has the potential to impact their own health and health-related decisions as they face age-related health declines themselves [84]. While Baby Boomers are better informed about health than previous generations, many factors impact their health decisions and health behaviors. One study of Baby Boomers' perceptions of hypertension found that they are concerned about the condition, yet reported poor blood pressure control and non-adherence to treatment [85]. In addition, health disparities among minority and low-income groups will continue into old age, resulting in poorer health and disability for these groups. Whether the Baby Boomer generation will have the same, more, or fewer health issues in later life than prior generations, as well as factors that can positively influence these outcomes, including use of CHIT, is unclear.

### 3.4. External ecological influences

An ecological perspective on Baby Boomers' CHIT use and PHIM behaviors draws attention to various nested levels of influence in which individual level CHIT use and PHIM behaviors occur. These influential factors include family and professional relationships, organizational and community settings, and social, cultural and political factors. Such contexts provide distinguishing features or facilitating factors for PHIM in Baby Boomer subgroups. For example, Baby Boomers in the US may be living independently, with a significant other, in a multi-generation family context, or contemplating changes in living arrangements as they retire. Their health information sources may include friends and family, employer initiatives (e.g. corporate wellness programs), insurance company incentives (e.g., Silver Sneakers fitness programs), nonprofit organizations (e.g. American Heart Association), and community resources (e.g. OASIS Older Adult Service and Information System).

Additionally, as previously stated, an ecological perspective considers the influence of numerous socio-cultural factors of importance for CHIT use and PHIM behaviors. An understanding of the socio-cultural context of Baby Boomers' health as influenced by the health care system, the health information and technology environment, interpersonal communication, and family relationships is imperative. For example, a patient may be less likely to comply with physician recommendations for home care that requires rest if the patient has little familial support. Similarly, a person in need of social support for a chronic illness who has work responsibilities that preclude involvement in face-to-face support groups might engage in online support groups. Therefore, the underpinning to this proposed framework is the ecological perspective that interpersonal, socio-cultural, and technological factors are interconnected in complex ways that are meaningful for understanding CHIT use for PHIM purposes. Collectively, such contexts are considered as external influences important when examining Baby Boomers' PHIM using CHIT.

One external influence of importance for consideration is that of health literacy. Scholars working in the area of health literacy have recently reframed the issue from an individual level problem to a system level one. That is, the health care organizations and systems are incredibly complicated and hard to understand, which complicate challenges for those limited in obtaining and using health information to make prudent health decisions. Scholars argue for transforming health care organizations and systems so that health information is easy to access and understand [86],[87] CHIT has an important role to play in this transformative work.

Additional external influences stem from relationships with those relevant to Baby Boomers' PHIM. Examination of patient-physician interaction as it relates to information seeking and technology is particularly important for understanding Baby Boomer PHIM and CHIT behaviors. The advent of the Internet and a better-informed health consumer has impacted the patient-practitioner relationship [65]. Studies demonstrate that actively searching for health information online after a physician visit is associated with lower trust and disappointment with some aspect of the physician's services [88]. Discussing information found from the Internet with a healthcare provider may serve the purpose of assessing the capabilities of the provider, which can be indicative of provider trust issues and might have implications for compliance with treatment [89]. Although primary care physicians are an important source of health information, Baby Boomers frequently experience distrust of their doctors [90] and thus may turn to alternative sources of health information such as Internet [65],[88]. Technology introduces changes to the communication process between the doctor and the patient and the roles of providers, which may affect health outcomes if not recognized and managed [65].

Other interpersonal relationships also have meaningful influences on Baby Boomers' PHIM. Marital and romantic relationships have implications for health support and management. As suggested previously, increased rates of divorce and remarriage among Baby Boomers result in complex emotional, legal, and financial demands unique to their generation. Additionally, these varied familial patterns impact Baby Boomers' health support as they disrupt intergenerational bonds and decrease a child's sense of filial obligation to assist older family members [91]. Where previous generations may have enjoyed (and continue to enjoy) support from their adult children when they encounter health declines, Baby Boomers may not receive the same support. Relationships with siblings and friends may be important sources of health information and support that could offset Baby Boomers' challenges in personal health management. Baby Boomers have experienced longer relationships with siblings than earlier or later cohorts [92]. Siblings share a cultural and physiological background and might provide valuable insights for PHIM, as many friends who are of similar age and enjoy mutual respect [93]. Although the influence of interpersonal relationships on health, gathering of health information, and health management is clear, the extent to which such ties could be influenced and managed through CHIT is not well understood.

## 4. Discussion—Future directions and call for research

CHIT tools, particularly those that facilitate information seeking, finding, and sharing, may help address the needs of Baby Boomers. However, research to date has not adequately explored the degree to which Baby Boomers are ready to embrace CHIT and how their unique subcultures affect adoption and diffusion. This paper provides support for advancing an ecological model of Baby Boomers' PHIM using CHIT to guide future research addressing Baby Boomers' PHIM behaviors. To set the foundation for future work, this paper calls attention to what the literature tells us about how Baby Boomers access, retrieve, organize, integrate, track, and use personal health information to make healthcare related decisions and perform health behaviors.

Due to the “anytime and anywhere” nature of PHIM behaviors, multiple types of health information tend to be fragmented and scattered in multiple repositories and media [41],[59],[94]. Further, individuals may need to manage their personal health information among various relevant others, such as family members who participate in care management. These characteristics of personal health information produce “information problems” that consumers face when trying to manage their personal health information. These problems are compounded by the fact that personal health information is hard to a) collect and record, b) track, c) integrate, d) access and retrieve, and e) understand and use due to overload and complexity of the information.

These information problems are particularly salient for Baby Boomers' PHIM. By reviewing the literature on Baby Boomers' PHIM behaviors, some characteristics of PHIM behaviors of this age group can be summarized as follows. Baby Boomers:

- access their personal health information by actively seeking information or passively receiving information,
- weave their own information web depending on their disease trajectory,
- utilize personal relationships to help them understand and use this information,
- desire a relationship with a health care professional to cope with complicated and sometimes conflicting information [95].

These characteristics have important implications for Baby Boomers' management of PHIM information problems.

Information overload and explosion challenges Baby Boomers to sift through large quantities of information as well as decipher the quality of the information sought [46]. How to evaluate health information on the Web and find credible online health information are critical issues for Baby Boomers. Baby Boomers use online information to assist with health related decisions and self-management of chronic diseases. When it comes to wellness, Baby Boomers use online health information to seek fitness advice, enhance daily well-being, and take preventive actions. Although quite a few studies have been published about evaluation of online health information, Baby Boomers' use of such criteria or guidelines has been little studied and warrants further exploration.

**Figure 3.**
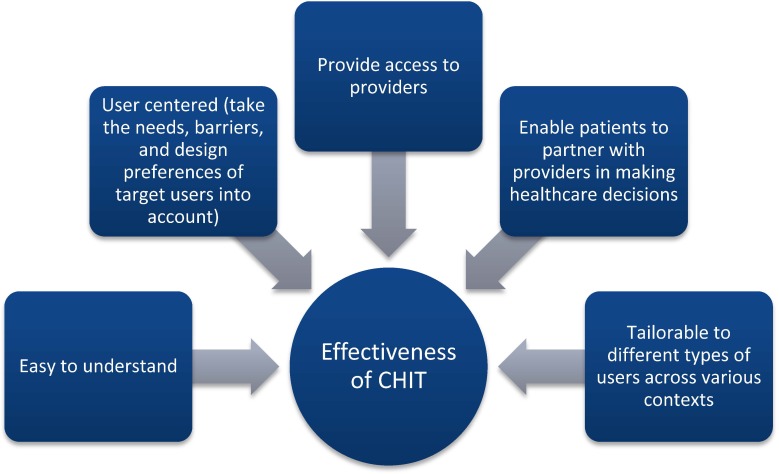
When CHIT Works Best (model inspired by Kreuter, et. al.) [96].

In summary, CHIT tools can facilitate how well Baby Boomers assume or continue health-enhancing behaviors and create health-promoting contexts. Figure 3 provides a model inspired by past literature indicating when CHIT tools work best.

However, current CHIT may not address these factors. Limited persuasive design considerations combined with technological constraints in sensing opportune moments for influence may be contributing to the failure of health applications to motivate people to change their health care behaviors [97]. To realize the potential that modern technologies have for Baby Boomers, foundational work to promote meaningful and effective health information seeking, storage, sharing and use by this target group must be done. This will be achieved through careful assessment and consideration of the challenges and needs of Baby Boomers in order to determine the features and design of the technology platforms and systems for sharing and exchange that best meet their needs. Further work is needed to conduct just such an assessment and establish the evidence base for health information system and application design to prepare for the growing and rapidly aging Baby Boomer population. Future research is called for in a number of areas relating to CHIT and PHIM at the individual and health care systems level, and from a design standpoint.

The following three tables (Tables 1 through 3) explicate research areas and examples of associated research questions. Research opportunities centered on Baby Boomers' individual-level factors associated with CHIT and PHIM are presented in Table 2.

**Table 1.** Potential Research Areas: Baby Boomers, CHIT and PHIM.

Potential Research Areas to Explore	Examples of Associated Research Questions
The characteristics of Baby Boomers that influence their use of CHIT and PHIM, as well as more in-depth information on Baby Boomers' practices related to CHIT and PHIM.	What Baby Boomer CHIT access issues exist for PHIM? What health literacy issues do Baby Boomers face in using CHIT and PHIM? What Baby Boomer subgroups have difficulty using technology to its full potential? What are the training needs of Baby Boomers and who is poised to provide this training?Are there salient differences in CHIT or PHIM needs or preferences in terms of demographic characteristics (gender, age), social and cultural factors (immigrant groups, minority groups) or in terms of socio-economic status or varying health literacy?
The needs and preferences of Baby Boomers for different communication technologies as they relate to health care.	How do the needs and preferences of Baby Boomers for different communication technologies compare to digital natives and the aged? What are the barriers to Baby Boomers for using specific technologies for information seeking purposes?
The effect of the different forms of CHIT on interactions with health care professionals and systems on PHIM.	How should providers interact with Baby Boomers regarding information seeking? Do Baby Boomers require rich media communication to feel satisfied with their interactions with providers? What opportunities do different forms of CHIT offer for enhancing Baby Boomers' interactions with their physicians and provider-patient relationships? What constraints are associated with the use of CHIT interactions between Baby Boomer patients and their providers? How do Baby Boomers' perceptions of the effect of CHIT on physician-patient interactions differ from their providers' perceptions?
The way Baby Boomers navigate an information-rich environment in a manner that does not lead to information overload.	How do Baby Boomers with high needs for information differ from Baby Boomers with low needs for information? What training and systems are needed to prevent information overload? How do Baby Boomers handle information overload?
The needs and preferences of Baby Boomers for storing and organizing their personal health information.	How do Baby Boomers store and organize their personal health information? What training and systems are needed to support organization of PHI?
The avenues and preferences of Baby Boomers for sharing personal health information through online avenues or their traditional face-to-face social network, or other.	How do Baby Boomers decide with whom to share what health information? What features do Baby Boomers desire to ensure privacy of their health information (e.g., what personal health information should be shared, with whom, at when, and for what purpose)? What are the interactive influences between their social networks, both online and face-to-face, and health information sharing activities?
The way Baby Boomers use the information they gather to make health decisions (e.g., care, preventative measures).	What messages and connections are needed to help Baby Boomers make good decisions regarding their health? What types of CHIT content is most useful for decision making after diagnosis of a chronic condition?

It is important that we understand the role of external ecological influences on CHIT and PHIM for Baby Boomers. Many questions still need to be answered as shown in Table 3.

**Table 2.** Potential Research Areas: External Ecological Influences in CHIT and PHIM.

Potential Research Areas to Explore	Examples of Associated Research Questions
The current and potential roles of healthcare providers (assessment of technologies, influencing patient use, and potential secondary user) in facilitating Baby Boomers' use of CHIT for their PHIM.	What role can health care providers play in helping consumers not only access information but find accurate and usable information? How would different attitudes and roles of healthcare providers in CHIT adoption impact the patient-provider relationship? How can CHIT and PHIM applications advance desired health system practice goals, (e.g. patient-centered medicine, shared decision making) and outcomes (e.g. improved adherence, morbidity/mortality, and patient satisfaction)?
The current and potential roles of health organizations (processes, protocols and procedures, providing supporting infrastructure) in facilitating Baby Boomers' use of CHIT for their PHIM.	How do health care organizational policies, procedures, and protocols, support or hinder Baby Boomers' use of CHIT for their PHIM? What role should health organizations play in developing CHIT for PHIM for Baby Boomers? What role should health care organizations play in financially supporting development and use of CHIT by Baby Boomers?
The current and potential roles of a health consumer's health support network (family and friends) in facilitating Baby Boomers' use of CHIT for their PHIM.	How do Baby Boomers with children (i.e. digital native offspring) differ in their perceptions and use of CHIT for PHIM? How does a caretaking role (caring for aging parents) influence Boomer's perceptions and use of CHIT for PHIM?

Additional information is needed to design information and platforms that support information distribution that is accessible and usable by an aging Baby Boomer population.

**Table 3.** Potential Research Areas: Designing Information and Platforms.

How Baby Boomers might be segmented in terms of design, feature, functionality and media preferences for CHIT and PHIM.	What technologies are Baby Boomers ready to use for PHIM? How do the technology tools that Baby Boomers report they are ready to use for PHIM differ from the technology tools younger adults and older adults report that they are ready to use for health purposes? What barriers exist for Baby Boomers using various forms of technology tools for PHIM?
Given that significant numbers of Baby Boomers already have chronic conditions or have risk factors for developing them, how can communication technology developers and entrepreneurs intervene to improve health management and support successful aging for the Baby Boomer population	What CHIT interventions need to be developed and tested to help Baby Boomers maintain healthy lifestyles and prevent or manage chronic health conditions? What health outcomes can be improved by these interventions?
System constraints and CHIT design	What health system characteristics (policies and practices) constrain or enhance design and adoption of CHIT and PHIM? How can CHIT and PHIM protocols be designed to best address or fit health system strengths and weaknesses?

## 5. Conclusion

As the Baby Boomer generation moves into old age, technology holds promise for improving the health and successful aging of this large and diverse group and preventing escalating costs to the health care system. Further research is needed to understand their characteristics, needs, preferences and problems related to the use of this technology. The model proposed in this paper provides a framework for this future research by identifying the important components that we need to understand in order to promote the successful use of technology to impact health outcomes for this important population. It is hoped that this model and the associated, specified potential research areas to explore will encourage continued conversation regarding these components and research in this area so that this promise can be achieved.
